# Supplementary material for: Sexual Risk Behavior and Lifetime HIV Testing: The Role of Adverse Childhood Experiences
Source: Int J Environ Res Public Health. 2022 Apr 5;19(7):4372. doi: 10.3390/ijerph19074372 (PMC8998687; doi:10.3390/ijerph19074372)
Supplement: Supplementary file 1 [file ijerph-19-04372-s001.zip › ijerph-1617618-supplementary.pdf]

**Supplement 1.** Unadjusted and adjusted prevalence ratios (and 95% confidence intervals) for the association between sexual and gender identity and self-reported HIV risk (n=58,077)		
	**Unadjusted**	Adjusted*
**Sexual and Gender Identity**		
Cisgender Bisexual Women	**3.24 (2.43, 4.32)**	1.05 (0.82, 1.34)
Cisgender Bisexual Men	**3.49 (2.39, 5.07)**	1.46 (0.96, 2.21)
Cisgender Gay Women	0.84 (0.37, 1.94)	0.41 (0.25, 1.02)
Cisgender Gay Men	**5.86 (4.49, 7.64)**	**2.92 (2.21, 3.85)**
Cisgender Heterosexual Women	**0.59 (0.50, 0.69)**	**0.62 (0.52, 0.73)**
Cisgender Heterosexual Men	**Reference**	**Reference**
Cisgender Other Sexual Identity Women	1.40 (0.70, 2.80)	0.84 (0.40, 1.75)
Cisgender Other Sexual Identity Men	1.53 (0.84, 2.80)	0.89 (0.49, 1.58)
Cisgender Questioning Women	0.77 (0.23, 2.55)	1.19 (0.34, 4.15)
Non-Binary	0.69 (0.22, 2.18)	0.66 (0.23, 1.89)
Transgender Women	2.58 (0.96, 6.94)	1.20 (0.57, 2.51)
Transgender Men	1.72 (0.42, 7.00)	1.09 (0.33, 3.62)

Adjusted for the ACEs index, income, age, highest education level, race/ethnicity, depression, and binge drinking. Cisgender questioning men not included in analyses due to variance inflation limitations.
